# The Relationship Between Early Maladaptive Schemas and Cluster C Personality Disorder Traits: A Systematic Review and Meta-Analysis

**DOI:** 10.1007/s11920-023-01439-3

**Published:** 2023-10-23

**Authors:** Angelos Panagiotopoulos, Akylina Despoti, Christina Varveri, Marie C. A. Wiegand, Jill Lobbestael

**Affiliations:** 1 Department of Psychology, National and Kapodistrian University of Athens, Athens, Greece; 2 Institute of Behavioural Research and Therapy, Athens, Greece; 3 Clinical Ergospirometry, Exercise and Rehabilitation Laboratory, 1st Intensive Care Department, School of Medicine, National and Kapodistrian University of Athens, Athens, Greece; 4 Clinical Psychological Science, Faculty of Psychology and Neuroscience, Maastricht University, University single 40, 6229 ER Maastricht, the Netherlands

**Keywords:** Early maladaptive schemas, Personality disorders, Cluster C, Schema therapy, Systematic review, Meta-analysis

## Abstract

**Purpose of review:**

We systematically reviewed and meta-analyzed the literature on the relationship between early maladaptive schemas (EMSs) and Cluster C personality disorders (PDs). Our aim was to clarify which of the 18 EMSs exhibit the strongest associations and are most frequently endorsed in clinical and non-clinical samples with Cluster C PDs and traits.

**Recent findings:**

After initially screening 2622 records, 12 studies were selected with 5310 participants. Meta-analyses of the raw correlation coefficients for each EMS-Cluster C PD link (3-8 studies per meta-analysis) indicated that the 18 EMSs were significantly related to all three Cluster C PDs with *r’s* ranging from .13 to .63. However, when considering endorsement rates among multiple regression studies that controlled for the EMSs intercorrelations and the effects of other PD traits and demographics, specific EMS constellations emerged for each Cluster C PD.

**Summary:**

Overall, the findings of the current paper suggest that Cluster C PDs might be conceptualized on the basis of a hybrid EMS model, in which all EMSs contribute to global personality dysfunction whereas specific EMS patterns reflect unique personality disorder style expressions. Longitudinal research with appropriate methodology is needed to draw more definite conclusions on the EMSs-Cluster C PDs relationships.

**Supplementary Information:**

The online version contains supplementary material available at 10.1007/s11920-023-01439-3.

## Introduction

Cluster C personality disorders (PDs) prevalence estimates range from 5.0% to 6.7% in the community [1, 2], to as much as 54% in psychiatric patients [3]. As conceptualized by DSM-5 [4], the cluster’s hallmark is self-related anxiety and fearful functioning, and comprises avoidant, dependent, and obsessive-compulsive PDs. Avoidant PD (AvPD) is characterized by excessive fears of criticism and rejection, social inhibition and restrain, feelings of inadequacy and inferiority, and oversensitivity to negative evaluation; dependent PD (DPD) by extreme fear of abandonment, submissive behaviours, and emotional dependence on others’ nurturance, reassurance, and guidance; and obsessive-compulsive PD (OCPD) by an inflexible fixation on orderliness, mental and interpersonal control, rules, and perfectionism.

In clinical practice, it is often assumed that Cluster C PDs are the least severe. Compared to other PDs, Cluster C PDs have received less empirical and clinical consideration [5]. For instance, several Cochrane reviews [6, 7] and meta-analyses [8, 9] have been published on borderline and antisocial PDs. At least eight international guidelines exist for the former and at least one for the latter [10]. In sharp contrast, best practice guidelines on Cluster C PDs are nonexistent and clinical therapy effectiveness studies are scarce [11]. This is problematic as their prevalence rates surpass those of Cluster B [1, 2] and—largely in contrast to several Cluster A and B PD patients—individuals with Cluster C PDs have a tendency to seek treatment [5, 12]. Furthermore, Cluster C PDs are linked to considerable functional impairment [13, 14] and socioeconomic burden [15, 16]. Evidence also suggests that Cluster C PDs have serious implications for the course of comorbid symptom-based disorders. Specifically, the prognosis of generalized anxiety disorder [17], social phobia [17, 18], obsessive-compulsive disorder [18], depression [19], and anorexia nervosa [20] is far worse when a Cluster C PD coexists. At the same time, compared to Cluster A and B, the risk of having a comorbid Cluster C PD is substantially higher across a range of mood [21], anxiety [22], and eating disorders [23]. Consequently, Cluster C PDs often remain undiagnosed, obscured by the conspicuousness of the comorbid symptom-based disorders for which patients tend to seek treatment [5]. In view of the above, calls have been made for more research attention on the diagnosis and treatment of Cluster C PDs [5, 13]. Accordingly, understanding how Cluster C PDs coincide with concepts in evidence-based treatment approaches for PDs appears to be a good first step in this direction. Here, we concentrate on the most fundamental concept of schema therapy—early maladaptive schemas (EMSs)—because schema therapy is one of the rare exceptions among the various approaches that has not almost exclusively focused on borderline PD but has expanded its application to most PDs, including those of Cluster C [24].

In schema therapy, EMSs lie at the core of longstanding characterological problems and PDs [25]. They are defined as dysfunctional, enduring, and pervasive patterns of information processing. They encompass explicit beliefs and memories at the conscious level as well as implicit knowledge, emotions, bodily sensations, and attention preferences about the self, others, and the world [25]. EMSs develop early in life in case core emotional needs are not met during childhood. This can result from the child’s aversive environment [26••], maladaptive parental rearing behaviors [27, 28], and/or insecure attachment [29], but also from the child’s temperament and character traits [30].

EMSs are gradually consolidated in long-term memory throughout one`s life [31], and eventually become automatically activated in response to external or internal stimuli, particularly those that show resemblance to the events that led to their development. When triggered, EMSs cause distress that is coped with through maladaptive strategies [30]. Ultimately, these coping strategies can cause pathological patterns including interpersonal problems [32••]. EMSs guide behavioral responses and distort information processing in such a way that it fits their content, which makes them self-perpetuating and rigid [33]. EMSs are also dimensionally associated with pathology; that is, their levels monotonically increase from healthy individuals to patients without PDs, to patients with PDs [34, 35•].

Young et al. [25] have suggested 18 different EMSs that are grouped into five domains (i.e., *Disconnection/Rejection*, *Impaired Autonomy/Performance*, *Impaired Limits*, *Other-Directedness*, *Over-Vigilance/Inhibition*) and are associated with five core emotional needs: attachment, autonomy, self-esteem, pleasure, and structure/limit setting. Although most clinical research has endorsed the 5-domain/18-EMS organization originally proposed by Young, psychometric evaluations have produced mixed results including a 4-domain/18-EMS model [36••], a bifactor model in which all 18 EMSs load onto a single general domain [37], or a 4-domain/20-EMS model [38]. So while recognizing that the overall findings on the primary and higher-order factor structure of EMSs are often conflictual (see also discussion), in the current study we use the 5-domain/18-EMS framework to stay methodologically consistent with clinical research and report all findings without having to systematically exclude relevant studies.

Although the relationship between EMS and PDs is well established [24], the specific EMSs–PDs associations remain rather unclear [33, 39•]. Regarding Cluster C in particular, studies have reported inconclusive results for all three PDs (for a more detailed discussion see [33, 39•]). For instance, a literature overview by Lobbestael and Arntz [33] indicated that hardly any EMS–Cluster C relation was reported by more than two studies.

These inconsistencies can probably be attributed to the great methodological heterogeneity among studies [33, 39•] in terms of study populations (including mixed patients, offenders, and convenient community samples); symptom severity (i.e., clinical and non-clinical samples); sample sizes (from *N* = 87 [40•] to several hundred [41•]); statistical analyses (e.g., bivariate correlations, regression analyses); and diagnostic tools to assess PDs (i.e., self-report instruments or semi-structured interviews). To our knowledge, no previous study has attempted to empirically review the literature on the topic, limiting the reliability of findings on the EMSs–Cluster C PDs associations. Clarifying these relationships would improve early detection, accurate diagnosis, and treatment outcomes of Cluster C PDs. By extension, it potentially improves the cost-effectiveness of healthcare systems [15, 42]. Therefore, the aim of the present study is to conduct a systematic review and meta-analysis of the current literature to clarify which EMSs are most strongly associated with Cluster C PDs/traits. Based on the PICO framework, the research question was formulated as follows: Which of Young’s 18 EMSs are most strongly linearly related to each of the three Cluster C PDs/traits as conceptualized in DSM-IV and DSM-5 in clinical and non-clinical adult samples?

## Method

This systematic review and meta-analysis was not pre-registered.

### Search Strategy

The search methodology followed the Preferred Reporting Items for Systematic Reviews and Meta-Analyses (PRISMA) statement [43]. It was conducted in the electronic databases of PubMed, Ovid Medline, Scopus, Web of Science Core Collection, Embase, and PsychArticles, and included studies published between January 1990 and August 2023. The bottom limit of 1990 was set because the Young Schema Questionnaire (YSQ) measuring EMSs was first published that year [44]. The following search terms were applied: (early maladaptive schem* OR schem* OR EMS OR Young Schema Questionnaire OR YSQ) AND (‘’personality disorder*’’ OR ‘’obsessive compulsive personality disorder*’’ OR ‘’obsessive compulsive trait*’’ OR ‘’OCPD’’ OR ‘’avoidant personality disorder*’’ OR ‘’avoidant personality trait*’’ OR ‘’dependent personality disorder*’’ OR ‘’dependent personality trait*’’ OR ‘’Cluster C’’). The search terms were applied to full texts, except for Scopus, where the terms had to appear anywhere in the title, abstract, or keywords. In Ovid Medline, Scopus, Web of Science Core Collection, and Embase additional inclusion (i.e., articles, articles in press, short surveys, and erratum) or exclusion (i.e., books, book chapters, book reviews, editorial material, letters, proceedings papers, meeting abstracts, review articles) criteria were applied beforehand in line with our selection criteria.

### Selection Criteria

The inclusion criteria were the following: (1) peer-reviewed journal articles and published dissertations reporting the linear relationship between EMSs and Cluster C PDs/traits; in which (2) EMSs were measured with any form of the YSQ; (3) Cluster C PDs/traits were measured by validated measures (self-reports or semi-structured interviews) based on DSM-IV or DSM-5 conceptualization; (4) EMS measures were administered before any exposure to an intervention; and (5) participants were at least 18 years of age. Furthermore, articles had to (6) report primary data; and (7) be written in English. Note that we did not include articles that only reported the relationship between schema *domains* and Cluster C PDs/traits because these lack specificity [28, 33].

### Young Schema Questionnaire

Various versions of the YSQ have been constructed. Based on clinical intuition, Young [44] first proposed the Young Schema Questionnaire-Long Form (YSQ-LF), compromising 205 items and reflecting 16 EMSs clustered in five higher-order categories (domains). The second edition of the YSQ-LF retained the 205 items but contained 15 EMSs [45], as one EMS (i.e., *Social Undesirability*) did not emerge as an independent factor in two factor analysis studies (for an extensive overview of the YSQ development see [46]). Young [47] also developed a 75-item short form (YSQ-SF), which captured 15 EMSs. Further modifications resulted in the final versions of both the YSQ long (YSQ-L3; 232 items; [48]) and short (YSQ-S3; 90 items; [49]) forms, extending the EMSs to 18.

### Data Extraction

Two researchers (PA, DA) screened all studies on their title and abstract. The full texts of all studies identified as relevant were read independently by both researchers, and evaluated against the eligibility criteria. There was a full consensus on the final eligible articles to be included. An additional manual search was performed on Google Scholar and on the reference list of all eligible studies to verify that no articles had been omitted by the database search. Relevant information from each eligible study was independently extracted by the two researchers into an electronic spreadsheet and collated by PA. The data extracted included information about the sample, study location, measures of the predictor and outcome variables, analyses, and control variables. Discrepancies were resolved by discussion.

### Quality

The methodological quality of each eligible study was evaluated using the 14 criteria of the Standard Quality Assessment Criteria for Evaluating Primary Research Papers [50]. Each criterion was scored on a three-point scale (2 = fully; 1 = partially; 0 = not meeting the criterion). If a criterion was not applicable to a certain study design, it was not scored. The final quality score for each eligible study was calculated by summing the total score of all applicable criteria and dividing it by the total maximum score [50], and ranged from 0 to 1 (i.e., lowest to highest possible methodological quality). Following Nicol et al. [51••] each paper’s quality was then categorized as ‘limited’ (≤0.5), ‘adequate’ (0.5> and ≤0.7), ‘good’ (0.7> and ≤0.8), or ‘strong’ (>0.8). Two researchers (PA, DA) assessed the quality of each study individually. Based on a mean-rating (k = 2), two-way mixed effects model, the intraclass correlation coefficient estimate for absolute agreement was .85, 95% CI [0.55, 0.95], indicating moderate to excellent interrater reliability [52].  A consensus meeting was held to resolve discrepancies. The cut-off score for exclusion based on methodological quality was ≤0.5.

### Analysis Plan

The data were analyzed in two steps. First, meta-analyses of the raw correlation coefficients were conducted for each EMS-Cluster C PD association using jamovi version 2.2 [53]. The analyses were carried out with the Fisher r-to-z transformed correlation coefficient as the outcome measure because this transformation helps to stabilize the variance and to normalize the distribution of correlation coefficients [54]. It was decided to use fixed-effects models for all analyses because (i) the aim of the meta-analysis was to summarize the results of the current literature and to investigate the average effects in the studies included in the analysis [54], (ii) random-effects models require five or more studies (current paper: 3-8 studies per meta-analysis) to consistently achieve powers that are greater than the power of the studies they are based on [55], and (iii) fixed-effects models allow drawing valid conclusions even in the presence of heterogeneity, as long as these are restricted to the set of studies included in the meta-analysis [54]. Cochran's *Q *test was used to assess the presence of between-study heterogeneity in effect sizes [56]. If statistically significant, variability in effect sizes cannot be entirely attributed to sampling error within studies. The *I*^2^ index was also used to assess the extent of between-study heterogeneity, with percentages of 25%, 50%, and 75% being considered as low, moderate, and high heterogeneity, respectively [57].

In the second step, we examined EMS endorsment in each Cluster C PD derived from regression analyses. This was deemed necessary as multivariate regression techniques are more suitable than correlation coefficients in revealing independent contributions, e.g., controlled for other EMSs, PD traits, and/or demographic variables [33]. Endorsement was defined as the ratio of studies that reported a significant EMS-Cluster C PD association to the total number of studies that assessed the given EMS-Cluster C PD. This ratio was expressed into percentage. For example, if *Emotional Deprivation*-AvPD was assessed by ten studies and four of them reported a significant relationship, *Emotional Deprivation* endorsement in AvPD was 40%.

## Results

**Fig. 1:**
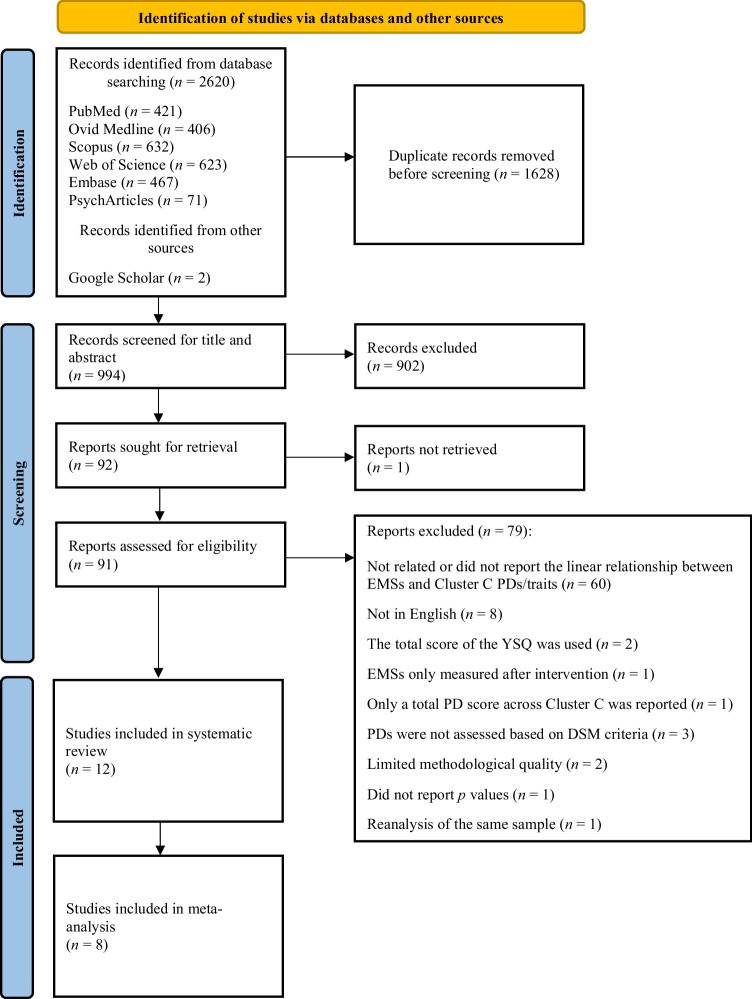
PRISMA flow diagram

The search initially yielded 2622 studies. Following the removal of 1628 duplicates, 902 were excluded based on the evaluation of their title or abstract. Ninety-two articles were sought for retrieval, yet one could not be accessed. After assessing the eligibility of 91 articles, a total of 15 were retained that adhered to the inclusion criteria. Another three studies were excluded from further analysis due to limited methodological quality [58, 59]; and because *p* values were not provided [60]. Further, two studies used the same sample [61•, 62], artificially inflating the results. We decided to keep only the former [61•] since it included all three PDs, in contrast to the latter that only reanalyzed AvPD in relation to childhood experiences [62].^1^ Finally, 12 studies were included with a total of *N =* 5310 participants (see Fig. 1 for Prisma flow diagram). Table 1 depicts the basic characteristics of these studies, while their raw findings can be found in Electronic Supplementary Material 1 (Table S1). One study conducted separate analyses for two independent samples [63•], and hence the samples are presented as two different studies in Table 1.^2^

**Table 1:** Overview of the studies included in the systematic review

Study & country	Sample type	Sample description	Cluster C PDs and EMSs assessment tools	Unit of EMS analysis	Analysis & control variables	Quality Rating
1. Bach et al. (2017) Denmark	Clinical	Mixed sample of outpatients and inmates *N* = 142 Age = 29.0 (*SD* not reported) 68.3% female	SCID-II, 90-item YSQ-S3	18 EMSs	Correlation, multiple regression Other EMSs (regression only)	Strong
2. Ball and Cereco (2001) USA	Clinical	Outpatients with substance use disorder *N* = 41 Age = 37.4 (± 5.9) 54% female	SCID-II, 205-item YSQ-LF	15 EMSs	Correlation No control variables	Good
3. Gilbert and Daffern (2013) Australia	Clinical	Convicted offenders *N* = 87 Age = 33.4 (± 10.7) 10% female	SCID-II, 90-item YSQ-S3	18 EMSs	Correlation No control variables	Strong
4. Kunst et al. (2020) Netherlands	Clinical	Mixed inpatient sample *N* = 130 Age = 43.6 (± 13.5) 51.5% female	SIDP-IV, 205-item YSQ-LF	15 EMSs	Correlation, multiple regression Age, education, gender, other PD traits, other EMSs (regression only)	Strong
5. Nordahl et al. (2005) Norway	Clinical	Mixed outpatient sample *N* = 82 Age = 37.7 (± 10.7) 56% female	SCID II, 205-item YSQ-LF	15 EMSs	Correlation No control variables	Good
6a. Pauwels et al. (2013) Sample 1^a^ Belgium	Clinical	Inpatients with eating disorder *N* = 218 Age = 21.70 (± 5.84) 100% female	ADP-IV, 205-item YSQ-LF-NL	16 EMSs	Multiple regression Age, other EMSs	Strong
6b. Pauwels et al. (2013) Sample 2 Belgium	Clinical	Inpatients with substance use disorder *N* = 351 Age = 44.57 (± 11.62) 31% female	ADP-IV, 205-item YSQ-LF-NL	16 EMSs	Multiple regression Age, gender, other EMSs	Strong
7. Steylaerts et al. (2023) Belgium	Clinical	Mixed inpatient sample *N* = 2043 Age = 39.63 (± 12.37) 47.2% female	ADP-IV, 75-item YSQ-SF-NL	15 EMSs	Partial correlation, hierarchical binary logistic regression Gender, general distress, categorical PD comorbidity (correlation and regression), other EMSs (regression only)	Strong
8. Thimm (2011) Norway	Clinical	Mixed outpatient sample *N* = 145 Age = 39.0 (± 11.9) 73.8% female	DIP-Q, 75-item YSQ-SF	Only EMSs hypothesised a priori for each PD AvPD: Defectiveness, Social Isolation, Failure, Subjugation, Insufficient Self-Control, Emotional Deprivation DPD: Dependence, Abandonment, Subjugation OCPD: Unrelenting Standards, Emotional Inhibition	Hierarchical regression Gender, big five personality traits, other hypothesized EMSs	Good
9. Bilge and Balaban (2021) Turkey	Non-Clinical	Community sample *N* = 654 Age = 33.23 (± 11.84) 56.4% female	CATI + TR, 90-item YSQ-S3-TR	14 EMSs	Correlation, multiple regression Other EMSs (regression only)	Adequate
10. Carr and Francis (2010) Australia	Non-Clinical	University students and people from the general community *N* = 178 Age = 27.18 (± 10.58) 66.3% female	PDQ4 + , 75-item YSQ-SF	15 EMSs	Hierarchical regression Gender, eating attitudes, depression, anxiety, other EMSs, comorbid PD symptoms	Good
11. Mącik (2018) Poland	Non-Clinical	Community sample *N* = 435 Age = 33 (*SD* not reported) 53% female	SCID-II Screen, 90-item YSQ-S3	18 EMSs	Multiple regression Temperament, parental attitudes, other EMSs	Good
12. Reeves and Taylor (2007) USA	Non-Clinical	University students *N* = 804 Age = 19.19 (± 1.44) 49.6% female	SCID-II Screen, 75-item YSQ-SF	15 EMSs	Multiple regression Gender, other PD symptoms from the same cluster	Strong

*ADP-IV* Assessment of DSM-IV Personality Disorders questionnaire, *CATI* + *TR* Coolidge Axis II Inventory Plus Turkish Short Form, *DIP-Q* DSM-IV and ICD-10 Personality Questionnaire*, PDQ4* + Personality Diagnostic Questionnaire – 4th edition, *SCID-II* Structured Clinical Interview for DSM-IV Personality Disorders, *SCID-II Screen* Structured Clinical Interview for DSM-IV Personality Disorders Screening Questionnaire, *SIDP-IV* Structured Interview for DSM-IV Personality, *YSQ-LF* Young Schema Questionnaire – Long Form, *YSQ-LF-NL* Young Schema Questionnaire – Long Form – Dutch Version, *YSQ-SF15-NL* Young Schema Questionnaire – Short Form – Dutch Version, *YSQ-S3* Young Schema Questionnaire – Short Form 3, *YSQ-S3-TR* Young Schema Questionnaire – Short Form 3 – Turkish Version, *YSQ-SF* Young Schema Questionnaire – Short Form

^a^Analyses were conducted for two sub-samples (patients with eating disorders and substance use disorders)

Across the 12 studies, various versions and language adaptations of the YSQ were employed. EMSs were measured by the 90-item YSQ-S3 [49] (*n* = 3), the 75-item YSQ-SF [47] (*n* = 3), and the 2^nd^ edition of the 205-item YSQ-LF [45] (*n* = 3). One study [64•] used the Turkish adaptation of the YSQ-S3^3^ [65], while Pauwels et al. [63•] and Steylaerts et al. [66•] employed the 16-factor Dutch versions of the YSQ-LF [67] and YSQ-SF^4^ [68], respectively.

In investigating the EMSs–Cluster C PDs relationships, all studies adopted a cross-sectional design. Eight studies (67%) used clinical samples (*n* = 3239; e.g., offenders, inpatients with an eating disorder, outpatients with substance use disorder), and four studies (33%) non-clinical samples (*n* = 2071; e.g., university students, people from the general community). Seven studies (58%) used self-report questionnaires and five studies (42%) semi-structured interviews to asses Cluster C PDs. Six studies (50%) were of strong, five (42%) of good, and one (8%) of adequate methodological quality.

### Meta-Analytic Findings

Of all reviewed studies, the raw zero-order correlations were available for eight studies [35•, 39•, 40•, 61•, 64•, 66•, 69•, 70•], which were included in the meta-analyses. The number of studies included in the individual meta-analyses ranged between 3 and 8, with 83.33% of the analyses being based on 5 or more studies providing data. For 87.04% of the analyses, the *Q* test was significant, suggesting that the true outcomes were heterogeneous. Based on the *I*^2^ index, the between-study heterogeneity was low for 9.26% of the meta-analyses, moderate for 14.81%, and high for 75.93%, with *I*² ranging from 0% to 94.22%. As shown in Table 2, all estimated average correlation coefficients were significant at *p* = .05 and remained significant at *p* < .0001 after applying the Bonferroni correction.

**Table 2:**
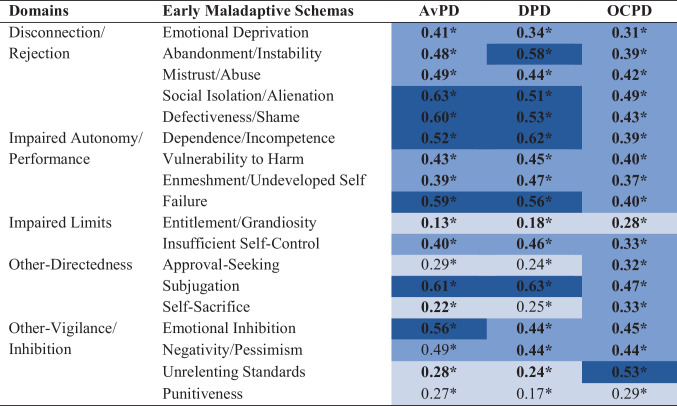
Estimated average correlation coefficients between Cluster C PDs and EMSs obtained from individual meta-analyses

Bold = true outcomes are heterogeneous (significant *Q* test). The color shade of the cells indicates the strength of the correlation coefficients according to Cohen’s (1988) guidelines, with light blue corresponding to small correlation size, light dark blue to medium size, and dark blue to large size

*EMSs* early maladaptive schemas, *AvPD* avoidant personality disorder, *DPD* dependent personality disorder, *OCPD* obsessive–compulsive personality disorder, *PDs* personality disorders

^*^ *p* < .0001

The correlation coefficients for the EMSs-Cluster C PDs relationships ranged from *r* = .13 to *r* = .63, corresponding to small to large correlations according to Cohen’s guidelines (small = .1, medium = .3, large = .5; [71]). Overall, all three Cluster C PDs tended to correlate most consistently and most strongly with EMSs in the domains of *Disconnection/Rejection* and *Impaired Autonomy/Performance*. While AvPD and DPD exhibited very similar patterns of correlations with all EMSs, some apparent differences emerged in comparison to OCPD. For instance, in the domain of *Other-Directedness*, AvPD and DPD both showed small correlations with *Approval-Seeking* and *Self-Sacrifice* and large correlations with *Subjugation*, while OCPD correlated moderately with all three of these EMSs. For the domain of *Other-Vigilance/Inhibition*, the picture was less consistent; while some EMSs (i.e., *Negativity/Pessimism* and *Punitiveness*) correlated similarly strongly with all three PDs, *Unrelenting Standards* showed a large correlation with OCPD but small correlations with AvPD and DPD, and *Emotional Inhibition* showed a large correlation with AvPD and medium correlations with DPD and OCPD. Within the domain of *Impaired Limits*, there were no such apparent differences between the PDs, with all three of them showing small correlations with *Entitlement/Grandiosity* and medium correlations with *Insufficient Self-Control*.

### EMS Endorsement as a Result of a Regression

EMS endorsement (as a percentage of significant associations) was assessed based on nine studies that used multivariate regression techniques [39•, 41•, 61•, 63•, 64•, 66•, 69•, 72•, 73•]. Six of these studies controlled for gender [39•, 41•, 61•, 63•, 66•, 72•], and four [39•, 41•, 61•, 66•] controlled for other PDs and/or other relevant symptoms (e.g., depression, anxiety). One study conducted logistic regression analysis with PD diagnosis as a binary outcome [66•], adopting a categorical perspective of personality pathology. The relevant findings are summarized in Table 3 (Tables S2-S4 in the Electronic Supplementary Material report in detail how these percentages were obtained for each Cluster C PD). Regarding AvPD, the EMSs of *Negativity/Pessimism, Emotional Inhibition,* *Social Isolation/Alienation,* *Failure,* and *Subjugation* stood out, followed by *Entitlement/Grandiosity* (negatively associated), *Approval-Seeking*, and *Defectiveness/Shame*. Correspondingly, in DPD the most prominent EMSs were by far *Dependence/Incompetence*, *Subjugation*, *Abandonment/Instability*, and *Enmeshment/Undeveloped Self*. Finally, *Unrelenting Standards* and *Self-Sacrifice* prevailed in OCPD. *Negativity/Pessimism* and *Punitiveness* were also prominent but were only assessed by two studies.

**Table 3:** EMS endorsement in Cluster C PDs as a percentage of significant associations derived from studies that used multivariate regression techniques

Domains	Early Maladaptive Schemas	AvPD	DPD	OCPD
Disconnection/Rejection	Emotional Deprivation	2/10 (20%)	0/8 (0%)	0/8 (0%)
	Abandonment/Instability	1/9 (11%)	**5/9 (56%)**	0/8 (0%)
	Mistrust/Abuse	2/9 (22%)	1/8 (13%)	1/8 (13%)
	Social Isolation/Alienation	**7/10 (70%)**	1/8 (13%)	2/8 (25%)
	Defectiveness/Shame	**3/10 (30%)**	0/8 (0%)	1/8 (13%)
Impaired Autonomy/Performance	Dependence/Incompetence	2/9 (22%)	**6/9 (67%)**	1/8 (13%)
	Vulnerability to Harm	2/9 (22%)^a^	0/8 (0%)	1/8 (13%)
	Enmeshment/Undeveloped Self	2/9 (22%)	**3/8 (38%)**	2/8 (25%)
	Failure	**5/10 (50%)**	2/8 (25%)	0/8 (0%)
Impaired Limits	Entitlement/Grandiosity	**3/9 (33%)**^**a**^	1/8 (13%)^a^	2/8 (25%)
	Insufficient Self-Control	2/10 (20%)	2/8 (25%)	2/8 (25%)^b^
Other-Directedness	Approval-Seeking	**1/3 (33%)**	0/2 (0%)	0/2 (0%)
	Subjugation	**4/9 (44%)**	**5/8 (63%)**	0/7 (0%)
	Self-Sacrifice	2/9 (22%)^a^	1/8 (13%)	**3/8 (38%)**
Other-Vigilance/Inhibition	Emotional Inhibition	**8/9 (89%)**	0/8 (0%)	2/9 (22%)
	Negativity/Pessimism	**3/3 (100%)**	0/2 (0%)	**1/2 (50%)**
	Unrelenting Standards	0/9 (0%)	0/8 (0%)	**8/9 (89%)**
	Punitiveness	0/3 (0%)	0/2 (0%)	**1/2 (50%)**

The percentages reflect how many statistically significant associations were found for each EMS among studies that included the respective EMS in their regression analysis. The most prominent percentages (i.e., ≥ 30%) are marked in bold

*EMSs* early maladaptive schemas, *AvPD* avoidant personality disorder, *DPD* dependent personality disorder, *OCPD* obsessive–compulsive personality disorder, *PDs* personality disorders

^a^Relationships were negative

^b^One study found a significant negative relationship

## Discussion

The present study investigated the relationship between EMSs and Cluster C PD traits, by systematically reviewing and meta-analyzing the current evidence base. The meta-analytic results showed that all 18 EMSs were significantly related to all three Cluster C PDs, exhibiting small to strong correlation coefficients. This pattern supports the notion that EMSs are dimensional trait-like constructs that constitute risk factors for psychopathology [74••], including personality pathology (e.g., [75]). Importantly though, EMSs are neither categorically related to PDs nor a simple descriptive taxonomy of PDs. Instead, they are also present in healthy individuals [25]. This overall pattern also points towards the high comorbidity and the underlying shared variance of PDs [76, 77], highlighting the limitations of a strictly categorical approach to PD pathology. The significant correlations of all 18 EMSs with all three Cluster C PDs likely also (partly) reflect a general severity factor of personality dysfunction [78–80], irrespective of the etiology (in our case EMSs) of this dysfunction [81]. For instance, emotional regulation impairment can occur on the basis of two seemingly opposing underlying EMSs; *Insufficient Self-Control* or *Emotional Inhibition*. Alternatively, our findings add that such a general severity factor of personality impairment might be more prominently captured by the EMSs in the domains of *Disconnection/Rejection* and *Impaired Autonomy/Performance* along with *Emotional Inhibition*, *Subjugation*, and *Negativity/Pessimism,* which tended to display the most consistent and strongest relationships across Cluster C PDs. In other words, the activation of these particular EMSs might interfere with general adaptive personality functioning—or in schema therapy terms with the healthy adult mode (see also [82••].

As opposed to these general EMSs-Cluster C PDs patterns, much more distinct EMS profiles emerged for all three Cluster C PDs when looking at EMS endorsement derived from regression studies. AvPD was characterized by eight EMSs, while DPD and OCPD by four. With the exception of *Entitlement* and *Approval-Seeking* in AvPD and of *Punitiveness* in OCPD, all highly endorsed EMSs among regression studies tended to consistently exhibit medium to large average correlation coefficients. Unifying the results from both types of analyses (i.e., meta-analytic and regression studies) raises two further points. First, Cluster C PDs might be best captured by a hybrid approach, whereby elevated scores across EMSs (especially within the domains of *Disconnection/Rejection* and *Impaired Autonomy/Performance*) reflect impaired personality functioning, but distinct EMS constellations give rise to unique Cluster C PD manifestations. Second, average zero-order correlations substantially inflate the magnitude and number of significant associations due to the high comorbidity of PDs [76, 77], the presence of other confounders such as gender [39•], and the high intercorrelations among EMSs [69•]. This is evident from the fact that some EMSs-Cluster C PDs relationships were of positive direction in the meta-analyses, but negative among regression studies (e.g., *Entitlement/Grandiosity* and *Self-Sacrifice* in AvPD). If we conceptualize EMSs as trait qualifiers of distinct personality style manifestations [82••]—in line with dimensional trait PD models [83]—specifying the traits of each PD in terms of EMSs requires controlling for the effects of other EMSs, other PD traits, and other potentially confounding variables. In this regard, EMS endorsement among studies using multivariate regression techniques constitutes the most useful way to unravel the unique EMS constellations of the three Cluster C PDs. In what follows, we mostly discuss the particular EMSs that seemed to be more prominent in each Cluster C PD in both types of analysis.

### Avoidant Personality Disorder

In AvPD the EMSs of *Negativity/Pessimism*, *Emotional Inhibition*, *Social Isolation/Alienation*, *Failure*, *Subjugation*, and *Defectiveness/Shame* stood out.^5^ The endorsement of the *Negativity/Pessimism* EMS is in line with evidence showing that avoidant traits are related to pessimism (e.g., [84]), negative interpretation, and attribution biases (e.g., [85, 86]). Likewise, *Defectiveness/Shame* and *Failure* are theoretically linked to AvPD [25, 87], and by definition, individuals with AvPD have low self-esteem and view themselves as unappealing and/or inferior to others [4]. A subsequent sense of being different and alienated from others and the world seems logical, as suggested by the endorsement of the *Social Isolation* EMS. This is further supported by research showing *Social Isolation* to be closely related to shame about characterological aspects of the self [88], which in turn contributes to experiencing loneliness [89], a feeling that goes beyond the mere perception of being alone [90].

It can be hypothesized that *Emotional Inhibition* and *Subjugation* are formed in an effort to cope with the EMSs discussed above. This is, to cope with negativity, social estrangement, and inner feelings of being defective and a failure, individuals with avoidant PD traits inhibit emotional expression and spontaneous action, and suppress their desires and needs to prevent being rejected, disapproved, or ridiculed. Indeed, Young et al. [25] posit that both EMSs are conditionally developed later in life to relieve distress caused by the unconditional EMSs (see also [91]). Further, Bamelis et al.’s [92] study showed that individuals with AvPD are characterized by transient coping states that are assumed to develop as survival strategies to alleviate the emotional pain caused by the affective state of being a lonely, inferior, and abused child—a state that contains many comparisons to the EMSs of *Defectiveness*, *Failure*, and *Social Isolation*. These coping states include detachment from inner needs, thoughts, and feelings (paralleling *Emotional Inhibition*), and compliance with others’ desires (paralleling *Subjugation*).

### Dependent Personality Disorder

The DPD prominent EMSs of *Dependence/Incompetence*, *Subjugation*, *Abandonment/Instability*, and *Enmeshment/Undeveloped Self,* nicely align with the theoretical notion that DPD comprises two independent forms of pathological dependency; emotional and functional [93, 94]. Emotional dependency is characterized by extreme fears of abandonment, beliefs about one’s inability to function in the absence of an intimate, nurturing relationship, and extreme needs for physical expressions of tenderness [93, 94]—features captured by the EMSs of *Abandonment/Instability* and *Enmeshment* (see also [95]). Indeed, emotional dependency is connected to dysfunctional attachment styles [93], consistent with the assertion that the *Abandonment/Instability* EMS is developed due to unmet needs for secure attachment [25]. Further, confirming Young et al.’s [25] hypothesis that *Subjugation* might build up in response to abandonment, emotionally dependent individuals surrender to others’ desires and suppress their own needs and emotions to avoid erratic behaviours or being abandoned by others.

In contrast, functional dependency mainly involves difficulties in decision-making, reluctance to take initiative to begin tasks independently, placing responsibility on others for important life areas, and constant seeking of guidance and reassurance due to lack of self-confidence and feelings of incompetence [93, 94]—aspects largely reflected by the EMS of *Dependence/Incompetence.* The conceptual overlap between functional dependency and the *Dependence/Incompetence* EMS is further accentuated by their common etiological pathway, since both are rooted in authoritarian and/or overprotective parenting [25, 93] that jeopardizes autonomous and competent functioning outside the family.

### Obsessive-Compulsive Personality Disorder

In OCPD, *Unrelenting Standards* was the most consistent EMS in both types of analysis. This seems reasonable considering that *Unrelenting Standards* reflects most DSM-5 OCPD criteria [4; p. 678–79]. According to Young et al. [25] this EMS is manifested as (i) perfectionism and unwarranted attention to detail (analogous to criteria 1 & 2), (ii) strict rules in life including moral precepts (analogous to criteria 4 & 8), and (iii) fixation to time and efficiency (criterion 3).

Our conclusions with regard to other EMSs in OCPD are somewhat less clear and should be regarded as tentative. *Self-Sacrifice* was frequently endorsed among regression studies, though the average correlation coefficient was relatively moderate in magnitude. *Negativity/Pessimism* and *Punitiveness* exhibited a high percentage of endorsement among regression studies but were only examined in two samples, while their average correlation coefficients were small to medium. *Self-Sacrifice* might be indicative of the tendency of individuals with OCPD traits to feel responsible for taking care of others, whom they consider needy, irresponsible, self-indulgent, and incompetent [96, 97]. Worthy of note, *Unrelenting Standards*, *Self-Sacrifice*, and *Punitiveness* have been recently clustered together under one latent factor—reflecting excessive responsibility and standards—which essentially characterizes OCPD [36••]. Finally, the EMS of *Negativity/Pessimism* presumably captures the belief commonly found in OCPD that the slightest flaw or mistake could prove catastrophic [96]. Hence, people with increased OCPD traits chronically ruminate, worry, and catastrophize about issues related to performance, organization, and control (e.g., mental, interpersonal, financial), which leads to indecisiveness and procrastination [98].

### Limitations and Future Perspectives

The current paper is not without limitations. First and foremost, the relatively small number of studies included in the meta-analyses coupled with high heterogeneity, raises issues on the precision of the estimated effect sizes [99]. Note though that the fixed-effects model we used is deemed statistically appropriate to aid a literature summary [54]. Second, the limited number of studies also prevented subgroup analyses to systematically assess moderating variables [100]. Three potentially moderating variables would be of major concern; the population (i.e., clinical and non-clinical), the type of PD assessment (i.e., self-report instruments and semi-structured interviews), and the YSQ version. Regarding this last point, it is important to mention that research on the primary and higher-order factor structure of the various YSQ versions employed across the included studies has produced heterogeneous and often psychometrically problematic results (e.g., 14 and 16 primary factors in the Turkish [65] and Dutch [67, 68] versions, respectively; several items cross-loading and/or not loading on any domain in the English version of the YSQ-L3 [38] and the Danish version of the YSQ-S3 [69•]). This raises concern over construct validity and hence, could have biased our results as the assumed latent variables (i.e., EMSs) across the 12 included studies might not be directly comparable (see [38, 101] for a detailed discussion on this issue). Third, considering that we restricted our inclusion criteria to the linear relationship between EMSs and Cluster C PDs traits, studies comparing mean EMS scores in Cluster C PDs to other disorders might have been overlooked.

Besides these limitations, our review points out many directions for future research on the EMSs-Cluster C relationships. First and most importantly, more research of sound methodology is needed to draw definite conclusions on the unique pattern of EMSs in Cluster C PDs. For instance, only a limited number of studies included in this review used the latest version of YSQ—which meant that information on the EMSs of *Approval-Seeking, Negativity*, and *Punitiveness* was very limited—or controlled for other PD traits and relevant symptoms (e.g., depression, anxiety). Ideally, future studies should (a) include large and mixed samples of healthy individuals and patients with different severity levels of Cluster C PDs; (b) use appropriate diagnostic instruments; (c) use the latest YSQ version which includes all 18 EMSs; and (d) employ multivariate analyses to control for other EMSs, PD traits, general psychopathology symptoms, and other potential confounders such as gender. Second, longitudinal research is necessary to establish whether and to what extent EMSs predict the onset of Cluster C PD symptomatology. In theory, EMSs are developed in childhood and predict the development of PDs in early adulthood [25], yet the cross-sectional design of the studies included in this review cannot provide any empirical indication of causality. Third, future studies examining EMSs in Cluster C PDs should consider new developments in schema theory such as the proposal of integrating three new EMSs [102••] and the existence of early *adaptive* schemas [103••]. For instance, the *Lack of a Coherent Identity* EMS [102••] might be pertinent in AvPD [104]. Finally, further research is warranted to unravel how EMSs in Cluster C PDs are related to schema modes, which reflect the temporary emotional-behavioural-cognitive states observed at one point in time [92, 105]. Importantly, schema modes found in Cluster C PDs—such as the abandoned child mode in AvPD and the self-aggrandizer mode in OCPD [92, 105]—are central to the treatment of personality pathology [106••].

## Conclusions

Overall, this review and meta-analysis found that all three Cluster C PDs were positively associated with all EMSs. This suggests that EMSs are related to general PD severity and personality functioning, and that PDs are better represented dimensionally rather than categorically. Nevertheless, specific EMS patterns also emerged for AvPD, DPD, and OCPD that were meaningful with regard to the diagnostic criteria, theory, and empirical evidence of these disorders. By extension, this implies that PDs could be conceptualized on the basis of a hybrid EMS model, whereby all EMSs might capture a global severity factor of personality dysfunction, whilst each PD is characterized by a distinct EMS constellation. However, additional longitudinal research with large mixed samples and sound methodology is necessary to completely unravel the EMSs-Cluster C PDs relationships.

### Supplementary Information

Below is the link to the electronic supplementary material.

Supplementary file1 (DOCX 21 KB)

Supplementary file2 (DOCX 22 KB)

Supplementary file3 (DOCX 22 KB)

Supplementary file4 (DOCX 22 KB)

## Data Availability

The data that support the findings of this study are available from the author upon reasonable request.

## Footnotes

1 Note that for the meta-analyses we extracted the correlation coefficients of Carr and Francis [61•] from their second (excluded) paper [62]

2 This study was not part of the meta-analysis; thus, it did not create issues of effect size dependency.

3 The Turkish YSQ-S3 contains 14 instead of 18 EMSs. Compared to the original YSQ-S3, this version does not include the Subjugation EMS, while six EMSs are merged into three factors: *Enmeshment/Undeveloped Self* with *Dependence/Incompetence*; *Entitlement/Grandiosity* with *Insufficient Self-Control*; and *Social Isolation/Alienation* with *Mistrust/Abuse*. In our analysis these three subscales of the Turkish version are considered as six separate EMSs.

4 The Dutch versions of the YSQ-LF and YSQ-SF incorporate the *Social Undesirability* EMS. However, it was excluded from further analysis because according to the latest advances in schema therapy, it is no longer considered a distinct factor [46].

5 *Approval-Seeking* also displayed a high percentage of endorsement among regression studies, yet it was examined in only three samples while at the same time it showed a small average correlation with AvPD. Hence, we cannot draw firm conclusions.
