# Supplementary material for: The Relationship Between Early Maladaptive Schemas and Cluster C Personality Disorder Traits: A Systematic Review and Meta-Analysis
Source: Curr Psychiatry Rep. 2023 Oct 23;25(10):439–53. doi: 10.1007/s11920-023-01439-3 (PMC10627891; doi:10.1007/s11920-023-01439-3)
Supplement: Supplementary file 1 — Supplementary file1 (DOCX 21 KB) [file 11920_2023_1439_MOESM1_ESM.docx]

**The Relationship Between Early Maladaptive Schemas and Cluster C Personality Disorder Traits: A Systematic Review and Meta-Analysis**

Current Psychiatry Reports

Angelos Panagiotopoulos^1,2^, Akylina Despoti^3^, Christina Varveri^2^, Marie C. A. Wiegand^4^, Jill Lobbestael^4^

^1^Department of Psychology, National and Kapodistrian University of Athens, Athens, Greece

^2^Institute of Behavioural Research and Therapy, Athens, Greece

^3^Clinical Ergospirometry, Exercise and Rehabilitation Laboratory, 1st Intensive Care Department, School of Medicine, National and Kapodistrian University of Athens, Athens, Greece

^4^Clinical Psychological Science, Faculty of Psychology and Neuroscience, Maastricht University, Maastricht, the Netherlands

**Contact: Jill Lobbestael,** Clinical Psychological Science, Faculty of Psychology and Neuroscience, Maastricht University, University single 40, 6229 ER Maastricht, the Netherlands. [Jill.lobbestael@maastrichtuniversity.nl](mailto:Jill.lobbestael@maastrichtuniversity.nl)

| **Table 1S** Overall findings of the studies included in the systematic review | | | |
| --- | --- | --- | --- |
| Study | EMSs related to AvPD traits  (ranked from highest to lowest r) | EMSs related to DPD traits  (ranked from highest to lowest r) | EMSs related to OCPD traits  (ranked from highest to lowest r) |
| 1. Bach et al. (2017) | Failure, Self-Sacrifice (neg), Emotional Inhibition, Social Isolation, Defectiveness, Subjugation, Emotional Deprivation, Insufficient Self-Control, Dependence, Negativity (*p* < .001) | Dependence, Subjugation, Abandonment, Enmeshment, Failure, Insufficient Self-Control (*p* < .001) | Unrelenting Standards, Enmeshment, Punitiveness, Self-Sacrifice (*p* < .001) |
| 2. Ball & Cereco (2001) | Subjugation (*p* < .05) | N/A | N/A |
| 3. Gilbert & Daffern (2013) | Defectiveness, Emotional  Inhibition, Emotional Deprivation, Failure, Social Isolation, Subjugation, Negativity, Punitiveness, Vulnerability to Harm, Mistrust, Abandonment, Dependence (*p* < .001) | Subjugation (*p* < .001) | No significant associations |
| 4. Kunst et al. (2020) | Correlation & regression analyses: Emotional Deprivation, Defectiveness, Social Isolation (*p* < .001), Failure, Emotional Inhibition, Abandonment, Mistrust, Enmeshment (*p* < .05)  Correlation analysis only:  Subjugation, Dependence, Self-Sacrifice, Insufficient Self-Control (*p* < .001), Unrelenting standards (*p* < .05) | Correlation & regression analyses: Self-Sacrifice, Subjugation (*p* < .001), Enmeshment, Insufficient Self-Control (*p* < .05)  Correlation analysis only: Abandonment, Failure, Dependence, Defectiveness, Emotional Inhibition, Unrelenting Standards (*p* < .001), Social Isolation, Mistrust (*p* < .05) | Correlation & regression analyses: Unrelenting Standards, Entitlement (*p* < .001), Emotional Inhibition, Dependence, Social Isolation (*p* < .05)  Correlation analysis only:  Insufficient Self-Control, Self-Sacrifice, Defectiveness, Subjugation, Failure, Enmeshment, Vulnerability to Harm (*p* < .05) |
| 5. Nordahl et al. (2005) | Social Isolation, Failure, Subjugation (*p* < .001), Emotional Inhibition, Mistrust (*p* < .01) | Subjugation, Abandonment, Social Isolation, Defectiveness, Dependence (*p* < .001), Self-Sacrifice, Mistrust, Failure, Enmeshment (*p* < .01) | Emotional Inhibition, Insufficient Self-Control, Entitlement, Social Ιsolation (*p* < .001), Defectiveness (*p* < .01) |
| 6a. Pauwels et al. (2013)  Sample 1^a^ | Defectivenes (*p* < .01) | Dependence (*p* < .01) | Unrelenting Standards (*p* < .01) |
| 6b. Pauwels et al. (2013)  Sample 2 | Social Isolation, Emotional Inhibition (*p* < .01) | Dependence, Subjugation (*p* < .01) | Self-Sacrifice, Defectiveness, Unrelenting Standards (*p* < .01) |

| **Table 1S (continued)** | | | |
| --- | --- | --- | --- |
| Study | EMSs related to AvPD traits  (ranked from highest to lowest r) | EMSs related to DPD traits  (ranked from highest to lowest r) | EMSs related to OCPD traits  (ranked from highest to lowest r) |
| 7. Steylaerts et al. (2023) | Binary logistic regression analysis:  Emotional Inhibition, Social Isolation, Entitlement (neg), Insufficient Self-Control, Failure, Vulnerability to Harm (neg) (*p* < .001), Subjugation (*p* < .01)  Partial correlation analysis: Social Isolation, Emotional Inhibition, Failure, Defectiveness, Subjugation, Insufficient Self-Control, Emotional Deprivation, Entitlement (neg), Self-Sacrifice (neg) (*p* < .001), Vulnerability to Harm (neg), Abandonment (neg) (*p* < .01) | Binary logistic regression analysis:  Dependence, Abandonment, Subjugation (*p* < .001)  Partial correlation analysis: Dependence, Abandonment, Subjugation, Enmeshment, Unrelenting Standards (neg), Insufficient Self-Control, Failure, Entitlement (neg), Social Isolation (neg) (*p* < .001), Emotional Deprivation (neg) (*p* < .01), Mistrust (neg) (*p* < .05) | Binary logistic regression analysis:  Unrelenting Standards, Insufficient Self-Control (neg) (*p* < .001)  Partial correlation analysis: Unrelenting Standards, Self-Sacrifice, Insufficient Self-Control (neg), Enmeshment, Emotional Inhibition (*p* < .001), Entitlement, Subjugation (*p* < .01) |
| 8. Thimm (2011) | Failure (*p* <.05) | Subjugation, Dependence (*p* < .01) | Unrelenting Standards (*p* < .01) |
| 9. Bilge & Balaban (2021) | Correlation & regression analyses: Social Isolation/Mistrust, Failure, Negativity, Enmeshment/ Dependence, Emotional Inhibition, Approval-Seeking (*p* < .001)  Correlation analysis only: Defectiveness, Abandonment, Vulnerability to Harm, Emotional Deprivation, Entitlement/Insufficient Self-Control, Punitiveness, Self-Sacrifice, Unrelenting standards (*p* < .001) | Correlation & regression analyses: Failure, Abandonment, Social Isolation/Mistrust, Enmeshment/Dependence (*p* < .001)  Correlation analysis only: Defectiveness, Negativity, Vulnerability to Harm, Emotional Inhibition, Emotional Deprivation, Approval-Seeking, Entitlement/Insufficient Self-Control, Self-Sacrifice, Punitiveness, Unrelenting standards (*p* < .001) | Correlation & regression analyses: Social Isolation/Mistrust, Negativity, Entitlement/Insufficient Self-Control, Emotional Inhibition, Vulnerability to Harm (*p* < .001)  Correlation analysis only: Enmeshment/ Dependence, Abandonment, Defectiveness, Approval-Seeking, Failure, Emotional Deprivation, Unrelenting Standards, Punitiveness, Self-Sacrifice (*p* < .001) |
| 10. Carr & Francis (2010) | Subjugation, Entitlement (neg) (*p* < .01), Emotional Inhibition (*p* < .05) | Entitlement (neg) (*p* < .01), Abandonment (*p* < .05) | Unrelenting Standards (*p* < .01), Self-Sacrifice (*p* < .05) |
| 11. Mącik (2018) | Social Isolation, Emotional Inhibition (*p* < .001), Negativity, Subjugation (*p* < .01), Vulnerability to Harm (neg), Self-Sacrifice (neg) (*p* < .05) | N/A | N/A |
| 12. Reeves & Taylor (2007) | Social Isolation, Emotional Inhibition, Entitlement (neg) (*p* < .001) | Abandonment (*p* < .001) | Unrelenting Standards, Enmeshment (*p* < .001) |
| *Note*. EMSs=early maladaptive schemas; AvPD=avoidant personality disorder; DPD=dependent personality disorder; OCPD=obsessive-compulsive personality disorder; N/A=not applicable. All EMSs were positively associated to each PD, except if specified by ‘neg’. | | | |
