# Supplementary material for: The Relationship Between Early Maladaptive Schemas and Cluster C Personality Disorder Traits: A Systematic Review and Meta-Analysis
Source: Curr Psychiatry Rep. 2023 Oct 23;25(10):439–53. doi: 10.1007/s11920-023-01439-3 (PMC10627891; doi:10.1007/s11920-023-01439-3)
Supplement: Supplementary file 4 — Supplementary file4 (DOCX 22 KB) [file 11920_2023_1439_MOESM4_ESM.docx]

**The Relationship Between Early Maladaptive Schemas and Cluster C Personality Disorder Traits: A Systematic Review and Meta-Analysis**

Current Psychiatry Reports

Angelos Panagiotopoulos^1,2^, Akylina Despoti^3^, Christina Varveri^2^, Marie C. A. Wiegand^4^, Jill Lobbestael^4^

^1^Department of Psychology, National and Kapodistrian University of Athens, Athens, Greece

^2^Institute of Behavioural Research and Therapy, Athens, Greece

^3^Clinical Ergospirometry, Exercise and Rehabilitation Laboratory, 1st Intensive Care Department, School of Medicine, National and Kapodistrian University of Athens, Athens, Greece

^4^Clinical Psychological Science, Faculty of Psychology and Neuroscience, Maastricht University, Maastricht, the Netherlands

**Contact: Jill Lobbestael,** Clinical Psychological Science, Faculty of Psychology and Neuroscience, Maastricht University, University single 40, 6229 ER Maastricht, the Netherlands. [Jill.lobbestael@maastrichtuniversity.nl](mailto:Jill.lobbestael@maastrichtuniversity.nl)

| **Table 4S** Summary of findings on the relationship between EMSs and OCPD traits derived from studies that used multivariate regression techniques | | | | | | | | | | | | | |
| --- | --- | --- | --- | --- | --- | --- | --- | --- | --- | --- | --- | --- | --- |
| Domains | EMSs |  | | Studies | | | | | | | | Endorsement  per EMS | Endorsement  Within Domains |
|  |  | 1 | 4 | | 6a | 6b | 7 | 8 | 9 | 10 | 12 |  |  |
| Disconnection/ Rejection | Emotional Deprivation |  |  | |  |  |  | * |  |  |  | 0/8 (0%) | 4/40 (10%) |
|  | Abandonment/Instability |  |  | |  |  |  | * |  |  |  | 0/8 (0%) |  |
|  | Mistrust/Abuse |  |  | |  |  |  | * |  |  |  | 1/8 (13%) |  |
|  | Social Isolation/Alienation |  |  | |  |  |  | * |  |  |  | 2/8 (25%) |  |
|  | Defectiveness/Shame |  |  | |  |  |  | * |  |  |  | 1/8 (13%) |  |
| Impaired Autonomy/ Performance | Dependence/Incompetence |  |  | |  |  |  | * |  |  |  | 1/8 (13%) | 4/32 (13%) |
|  | Vulnerability to Harm |  |  | |  |  |  | * |  |  |  | 1/8 (13%) |  |
|  | Enmeshment/Undeveloped Self |  |  | |  |  |  | * |  |  |  | 2/8 (25%) |  |
|  | Failure |  |  | |  |  |  | * |  |  |  | 0/8 (0%) |  |
| Impaired Limits | Entitlement/Grandiosity |  |  | |  |  |  | * |  |  |  | 2/8 (25%) | 4/16 (25%) |
|  | Insufficient Self-Control |  |  | |  |  | neg | * |  |  |  | 2/8 (25%) |  |
| Other-Directedness | Approval-Seeking |  | ** | | ** | ** | ** | ** |  | ** | ** | 0/2 (0%) | 3/17 (18%) |
|  | Subjugation |  |  | |  |  |  | * | * |  |  | 0/7 (0%) |  |
|  | Self-Sacrifice |  |  | |  |  |  | * |  |  |  | 3/8 (38%) |  |
| Over-Vigilance/ Inhibition | Emotional Inhibition |  |  | |  |  |  |  |  |  |  | 2/9 (22%) | 12/22 (55%) |
|  | Negativity/Pessimism |  | ** | | ** | ** | ** | ** |  | ** | ** | 1/2 (50%) |  |
|  | Unrelenting Standards |  |  | |  |  |  |  |  |  |  | 8/9 (89%) |  |
|  | Punitiveness |  | ** | | ** | ** | ** | ** |  | ** | ** | 1/2 (50%) |  |
| *Note.* EMSs=early maladaptive schemas; OCPD=obsessive-compulsive personality disorder; neg = negative relationship. Each study has been numbered according to Table 1 of the main text; White cells indicate that the EMS was assessed and did not significantly relate to OCPD traits; Grey cells indicate that the EMS was significantly related to OCPD traits; * Not measured or not reported or excluded from the analysis by the authors; ** Not included in the Young Schema Questionnaire measure used by the authors. | | | | | | | | | | | | | |
